# Catastrophic health expenditure among households with differently abled children and adolescents in Puducherry, India: a mixed-method study

**DOI:** 10.3389/fpubh.2025.1647871

**Published:** 2025-09-22

**Authors:** Sona Priyanka Auroprem, Sitanshu Sekhar Kar, Loganathan Devaraj, Mahalakshmy Thulasingam

**Affiliations:** ^1^JIPMER International School of Public Health (JISPH), Jawaharlal Institute of Post Graduate Medical Education and Research, Puducherry, India; ^2^Department of Preventive and Social Medicine, Jawaharlal Institute of Post Graduate Medical Education and Research, Puducherry, India

**Keywords:** disability, OOPE, catastrophic health expenditure, health seeking, differently abled, compensating variation

## Abstract

**Introduction:**

Households with disabled members are at greater risk of catastrophic health expenditure (CHE) due to their continual medical visits and treatments, the consequences of which force them into vicious cycles of impoverishment and distress financing. Additionally, they face various challenges in accessing healthcare, which compromises Universal Health Coverage (UHC). A significant gap exists in the cost of health expenditure for differently abled children in India.

**Methods:**

A mixed-methods study was conducted among 192 households, comprising 96 with differently abled children and 96 without. Quantitative data on health expenditures, insurance coverage, and utilization of disability benefits were collected through structured interviews. Catastrophic health expenditure was defined using the 40% of Capacity to Pay (CTP) threshold. Additionally, 10 IDIs were conducted among households with differently abled children to explore barriers and facilitators to healthcare access.

**Results:**

Approximately 44.8% of the households experienced CHE at the 40% of CTP threshold. The most common type of disability reported was mental disability. Approximately 50% of the households did not have any insurance coverage, and only 44.8% were availing disability benefits for their child. As many as 77.1% of households experienced financial distress. A significant compensating variation was found. Barriers to health seeking included high medical expenses, poor accessibility, limited availability of specialized care, and lack of continuity in care. Facilitators such as good access to information, availability of healthcare facilities, and social support were also identified. Various recommendations to improve health-seeking were provided by parents.

**Conclusion:**

This study found a higher prevalence of CHE, distress financing, and numerous challenges for health-seeking among households with differently abled children, highlighting the need for prompt measures to address these issues.

## Introduction

Globally, it is estimated that approximately 1.3 billion individuals have some type of disability (1). According to Census 2011, in India, disabilities were most commonly related to movement (20%), seeing (19%), hearing (19%), and multiple disabilities (8%). The highest proportion of persons with disabilities was in the age group of 10–19 years (2). According to the World Health Organization (WHO), Out-of-Pocket Expenditure (OOPE) for health in India was estimated to be as high as 50.59% in 2020 (3). India’s Total Health Expenditure (THE) is estimated at 3.8% of the Gross Domestic Product (GDP). Underfunding of public sector facilities, combined with rapid expansion of private sector providers, has led to the escalation of OOPE healthcare (4). It is reported that approximately 30% of the Indian population, referred to as the “missing middle,” lacks monetary risk protection mechanisms (5). Households with differently abled members have been found to experience increased catastrophic health expenditure (CHE) in India, which is not in line with Sustainable Development Goal 3 that aims to attain Universal Health Coverage (UHC) (6, 7). In Puducherry, approximately 2.42% of the population lives with a disability, but only 55.4% possess a disability certificate, and less than half have health insurance coverage (2, 5).

Disability increases the likelihood of belonging to the two poorest quintiles by approximately 10%, and there is a troubling cycle of low education leading to poverty in the developing countries (8). Families with disabled children experience higher costs compared to their healthy counterparts, despite governmental provisions of financial aid (9, 10). These increased expenses are due to their frequent inpatient and outpatient visits, as well as prescribed medications (10). Households adopt various coping mechanisms, such as the use of savings, the sale of assets, borrowing, the utilization of current income, and a reduction in the consumption expenditure level. The consequence of these distress financing mechanisms varies according to the disease type, nature of the healthcare facility, insurance status, and financial stability of the household (11). Many disabled persons face difficulty in assessing healthcare benefits, and there has not been an adequate mechanism to assess the adequacy and coverage (12).

Healthcare challenges include a limited understanding of the referral system, insufficient rehabilitation programs, and issues with reliability and acceptability. Individuals with disabilities face additional barriers such as limited accessibility, stigma, discrimination, lack of specialized services, mental health concerns, social isolation, and financial constraints (13, 14). There is limited evidence from the region quantifying the magnitude of CHE among households with differently abled children. Our study aimed to find the proportion of households with differently abled children/adolescents incurring catastrophic health expenditure and the compensating variation (CV), and to explore the facilitators and barriers for seeking healthcare.

## Materials and methods

### Study design

A mixed-method, cross-sectional design was adopted, combining quantitative estimation of the prevalence and burden of CHE with qualitative exploration of lived experiences influencing healthcare access. The cross-sectional approach was appropriate for assessing the current magnitude of CHE and was feasible within the study’s time and resource constraints. The inclusion of qualitative interviews allowed cross-validation of findings and provided a deeper contextual understanding, thereby compensating for some of the inherent limitations of the cross-sectional design.

### Study setting

Puducherry, a Union Territory located on the southeastern coast of India, comprises four districts, with Puducherry district being the largest and most populous. The territory has a well-established primary healthcare network, with approximately 39 primary health centers (PHCs) across Puducherry, of which Puducherry district alone has approximately 32 PHCs, 55 sub-centers, and 611 Anganwadi centers providing community-based health, nutrition, and early childhood development services. Puducherry is also known for its numerous special schools and rehabilitation centers offering free or subsidized services for children and adolescents with disabilities, supported by both government schemes and non-governmental organizations. The relatively compact geography, high literacy rate, and accessibility to health services make Puducherry an important setting for public health research, particularly in community-based and disability-related studies.

### Study population

For the quantitative part, Group 1 included parents of differently abled children/ adolescents attending special schools, excluding households with more than one such child. Group 2 comprised households without differently abled children from selected Anganwadi areas, and in the qualitative part, we purposively interviewed parents from group 1 until data saturation. The study included households with a child or adolescent aged 0–19 years with any type of disability, who had been residing in Puducherry for at least 6 months. Households with more than one differently abled child and those unwilling to provide consent were excluded.

### Sample size calculations

Considering the 16% prevalence of CHE in India (15), with an absolute precision of 9%, a confidence level of 95%, a significance level of 5%, and a design effect of 1.5, owing to cluster random sampling, the sample size was calculated using Open Epi Version 3.0. Hence, 96 households with differently abled children/adolescents and 96 households without differently abled children/ adolescents for matching using propensity scores were involved in the study. The final sample size was 192.

### Sampling technique

A purposive sampling method was used to select participants for Group 1, as special schools maintain defined rosters of differently abled children. For Group 2, a multistage cluster random sampling approach was adopted. Five PHCs (two urban and three rural) were randomly selected from the 32 in Puducherry, in proportion to the urban and rural distribution of participants in Group 1. From each selected PHC, two Anganwadi centers were randomly chosen, and approximately 9–11 households per PHC were selected using computer-generated random numbers. If a selected household was locked, the next household on the list was approached for data collection.

### Study tools

A pre-tested, semi-structured questionnaire, adapted from validated National Sample Survey Office (NSSO) and WHO tools, was used to collect data. The questionnaire was reviewed by subject experts and refined based on feedback from a pilot study. An interview guide with semi-structured, open-ended questions was utilized to conduct in-depth interviews among parents from Group 1, with a sufficient number of probes included to comprehensively capture relevant information during the interviews. A pilot study was conducted among 10 households having a differently abled child/ adolescent, attending the special schools, and were excluded from the final study. This was done to test the feasibility, objectivity, and applicability of the questionnaire, and required modifications were made. A revised questionnaire was administered to the study population.

### Data variables and data collection

Parents of differently abled children were administered a questionnaire covering sociodemographic details, health expenditure, and wealth index. Direct medical and non-medical costs (consultation, investigation, drugs, diagnostics, travel, and food) were recorded with a 3-month and 6-month recall period for outpatient and inpatient care, respectively. To minimize recall bias, participants were encouraged to refer to medical bills, prescriptions, or discharge summaries, and interviewers were trained to probe chronologically. Data were then annualized to estimate OOPE and CHE. The parents without differently abled children/adolescents were addressed using the questionnaire containing only sociodemographic details and wealth index. To minimize selection bias and ensure comparability between groups, a 1:1 matching was performed using propensity scores. The propensity score for each participant was estimated based on key socio-demographic characteristics and the household wealth index. Each case was then matched to one control with a similar propensity score, thereby creating two groups that were statistically balanced with respect to these baseline variables. Post-matching household incomes were compared to estimate the additional income required by Group 1 to achieve a similar standard of living as Group 2. In-depth interviews were conducted among 10 purposefully selected parents from group 1 to explore the facilitators and barriers to health seeking. The final sample size was determined based on data saturation. In-person interviews were conducted by a trained public health trainee at the special schools, in seclusion, and at their convenience. An interview guide was used, and an audio recording was made after consent, and verbatim notes were taken. Each interview lasted 30–45 min, with no repeat interviews or dropouts. The summary points were discussed with participants for validation.

### Data analysis

Data were exported from Epicollect5 and analyzed using SPSS version 23. Categorical variables were summarized as frequency with proportions, with CHE reported alongside 95% CI, while continuous variables were expressed as median (IQR) or mean (SD) as appropriate. Health expenditure data were presented as median (IQR). The wealth index was constructed using Principal Component Analysis (PCA) on asset ownership variables, with items reduced based on their frequency, and households categorized into wealth quintiles. Propensity scores were generated via logistic regression, using sociodemographic variables and wealth quintiles, to match both groups for CV income analysis.

For the qualitative data, the audio recordings were transcribed verbatim and translated on the same day as the interview. A combination of content and thematic analysis was used to identify enablers, barriers, and recommendations for health seeking. NVivo software supported coding, which was reviewed by a second investigator to ensure reliability. Stakeholder involvement was ensured through feedback from parents. The COREQ checklist guided reporting standards, and the emerging codes were organized using a socio-ecological model. Integration of qualitative and quantitative findings was achieved using a joint display approach to meta-inference.

The Institutional Ethics Committee (IEC) approval was obtained before the study. Informed consent was obtained from the participants, and confidentiality was maintained throughout the study.

## Results

Table 1 presents the sociodemographic characteristics of the study households. The majority of children in both groups were aged 10-19 years, with a higher proportion of males in Group 1 and females in Group 2. Most heads of households had completed secondary education, and the majority were employed. A substantial proportion of households in both groups belonged to the lower socioeconomic strata, with more than three-fourths classified as below the poverty line. At the 40% capacity to pay (CTP) threshold, 44.8% (95% CI: 35.2, 54.7) of households with a differently abled child/adolescent were found to experience CHE, while with a decrease in the threshold to 10%, as high as 82.2% (95% CI: 73.5, 88.6) encountered CHE. The wealthiest quintile had the highest OOPE, with a median spending of ₹44,500, while the poorest quintile spent approximately ₹11,400 (Table 2). Rural households experienced more CHE (49.1%) than urban ones (39.5%). Distress financing was reported by 77% (95% CI: 67.7, 84.3) of households.

**Table 1:** Distribution of sociodemographic characteristics among households with and without a differently abled child/adolescent residing in Puducherry, India (*N* = 192).

Variable		Group 1 (*n* = 96), *n* (%)	Group 2 (*n* = 96), *n* (%)
Child’s age	Up to 5 years	20 (20.8)	23 (24.0)
	6–9 years	28 (29.2)	18 (18.7)
	10–19 years	48 (50.0)	55 (57.3)
Sex of the child	Male	64 (66.7)	38 (39.6)
	Female	32 (33.3)	58 (60.4)
Educational status of the head of the family*	No formal education	6 (6.3)	3 (3.1)
	Primary (1–8)	21 (21.9)	25 (26.0)
	Secondary (9–12)	50 (52.1)	44 (45.8)
	Undergraduate and above	19 (19.8)	24 (25.0)
Employment status of the head of the family	Employed	80 (83.3)	91 (94.8)
	Retired and unemployed	6 (6.3)	4(4.2)
	Homemaker	10 (10.4)	1 (1.0)
Socioeconomic status**	Upper (₹8,822 and above)	5 (5.2)	10 (10.4)
	Upper middle (₹4,411–₹8,821)	23 (24.0)	25 (26.0)
	Middle (₹2,647–₹4,410)	21 (21.9)	17 (17.7)
	Lower middle (₹1,323–₹2,646)	32 (33.3)	35 (36.5)
	Lower (<₹1,323)	15 (15.6)	9 (9.4)
Below poverty line (BPL)		75 (78.1)	78 (81.3)
Presence of disabled adults in the family		8 (8.3)	4 (4.2)

*Based on the Indian standard classification of education, ** modified BG Prasad scale.

**Table 2:** Average OOPE across the wealth quintiles among households with a differently abled child/adolescent, (*N* = 96).

Wealth quintiles*	OOPE (in INR)	
	Median	IQR
Poorest (Q1)	₹11,400	₹6,030, ₹15,800
Poorer (Q2)	₹16,360	₹8,800, ₹43,200
Middle (Q3)	₹20,200	₹6,800, ₹63,500
Richer (Q4)	₹15,825	₹2,637, ₹50,400
Richest (Q5)	₹44,500	₹7,350, ₹80,300

Awareness of the NIRAMAYA scheme (a government health insurance programme for persons with disabilities) was low, with only 24% of households aware of it and 8% enrolled. Distress financing, defined as borrowing, selling assets, or receiving contributions from friends/relatives to meet healthcare costs, was reported by 77% (95% CI: 67.7–84.3) of households with a differently abled child or adolescent. Travel costs were higher among rural households compared to urban households. Compensating variation represents the additional monthly income that households with a differently abled child would require to achieve the same living standard as a comparable household without a differently abled child. In our study, this figure was ₹3,000 (IQR ₹500, ₹12,375) per month.

Interviews were conducted with the parents of 10 children and adolescents with disabilities. The children included one aged 0–5 years, two aged 6–9 years, and seven aged 10–19 years. Six were male and four were female. Regarding the type of disability, there was one child each with hearing, speech, and visual impairments; four with mental disabilities; and three with locomotor disabilities.

### Barriers to health seeking

Parents of differently abled children face a multitude of barriers to health seeking, which include substantial healthcare expenses, including consultation fees and medical supplies. Apparently, they had to travel long distances for specialized services, leading to delayed and inadequate service utilization. The lack of effective communication strategies has made parents visit multiple healthcare facilities. Parents also expressed their concerns about poor prioritization and lengthy hospital waits. Social support was reported to be notably lacking. Many parents remain unaware of their child’s condition and available treatment options, making them vulnerable to scammers who sell unknown medicines. Some parents identified a lack of information as a barrier. Additionally, fear of being discriminated against has made parents avoid accessing welfare benefits, and it was reported that discrimination existed even among the parents of disabled children, based on the severity of the disability.

### Facilitators for health seeking

The availability of nearby healthcare facilities was reported to have enhanced timely access to care, encouraging health-seeking behaviour among parents and alleviating travel-related financial constraints. Parents are aware of early screening programs for proactive diagnosis and intervention. Parents have expressed that special schools have played a significant role in disseminating valuable healthcare-related information and providing aid. A robust social support mechanism within the society, including family, friends, and NGOs such as special schools, was acknowledged by the parents. The importance of disability benefits and various other schemes provided by the government and other NGOs was emphasized by the parents. The benefits, in addition to reducing financial stress for healthcare, also proved helpful in managing their daily livelihood.

### Recommendations from parents

**Figure 1:**
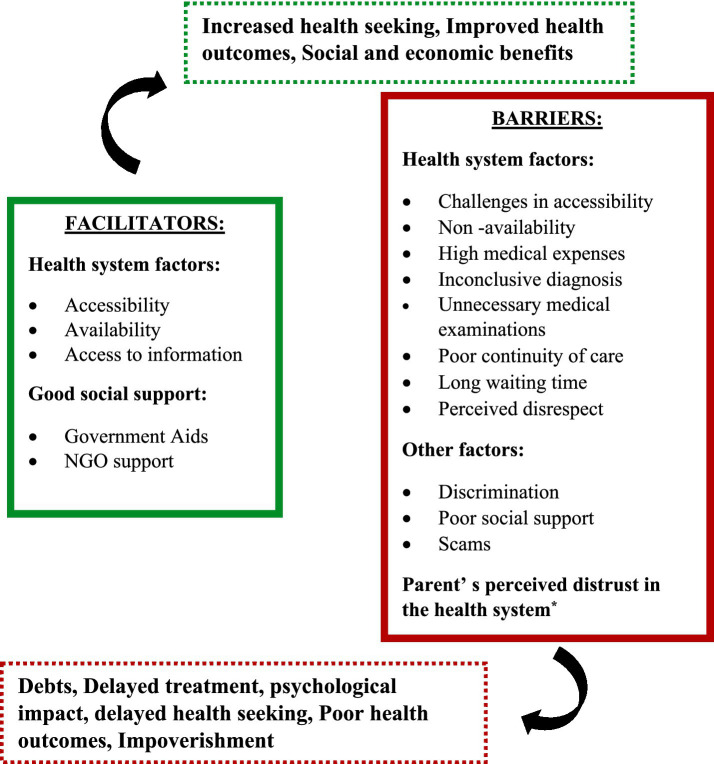
Factors influencing health seeking and their outcomes—an overview.

**Figure 2:**
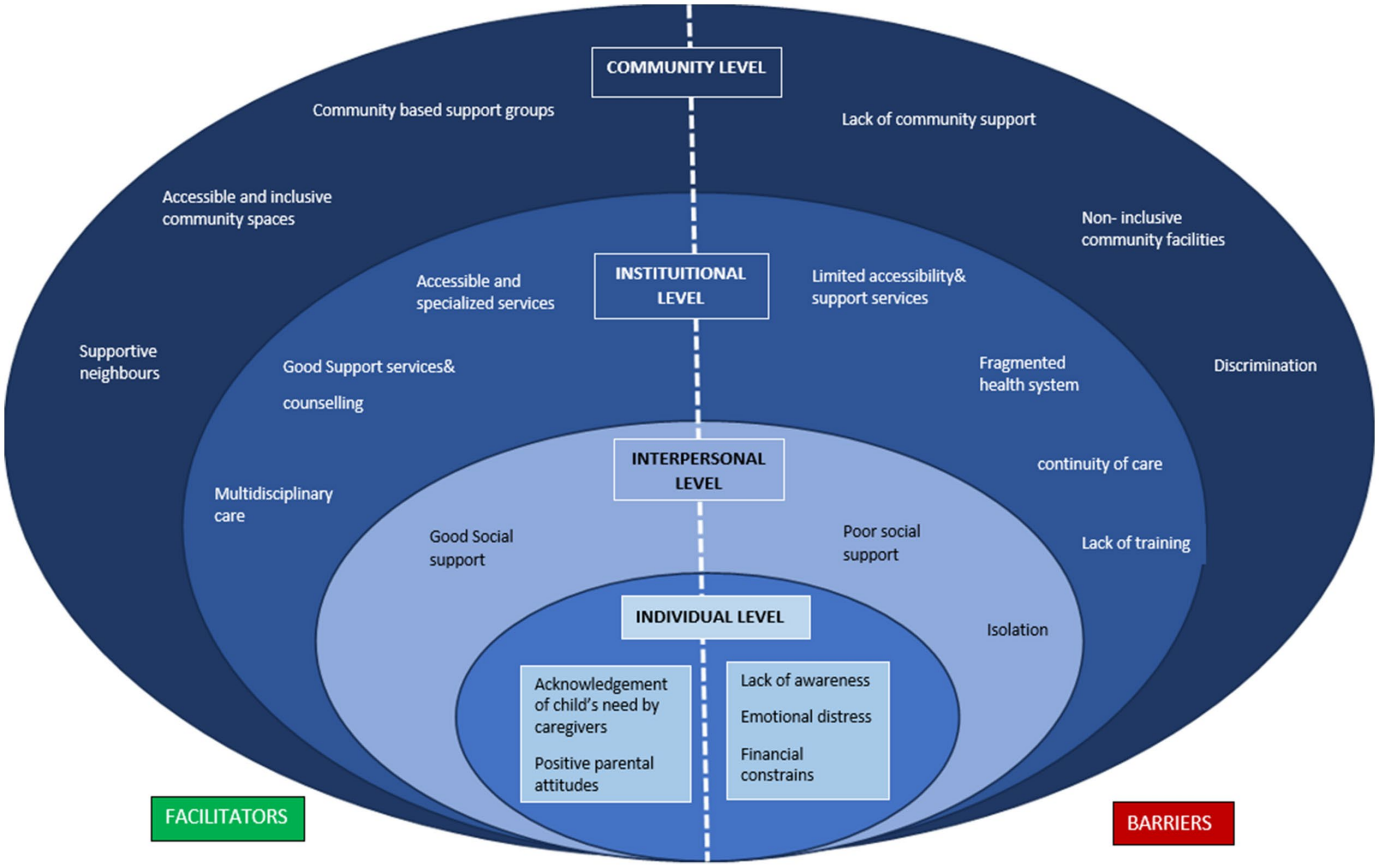
Socio-ecological model describing the facilitators and barriers for health seeking from the perspective of parents.

Parents gave various recommendations, such as organizing community-based camps, as they are exposed to numerous challenges pertaining to the transportation of their children to the hospital, and the added challenge of financial strain. Many parents recommended implementing an appointment system to reduce the wait time, as it is difficult to manage their children. They also advocated for the establishment of a special ward and hospital dedicated to disabled children and the provision of affordable diagnostics. They emphasized the lack of a proper referral and transparent communication mechanism in healthcare facilities and the prioritization of children with special needs. Parents advocated for offering diapers, rubber sheets, and other essential items at reduced prices, recognizing the financial strain families with disabled children often face. The figure depicts the socio-ecological model that has been adopted to describe the facilitators and barriers for health seeking from the parent perspective, at all various levels such as the individual, interpersonal, institutional, and community levels (see Figures 1, 2; Tables 3, 4).

**Table 3:** Themes, categories, and participant quotes on barriers, facilitators, and recommendations for health-seeking in households with differently abled children.

Themes	Categories	Verbatim quotes
Barriers for health seeking	High medical expenses	*“Spending for hospitals and medicines are our biggest problem. We spend money on regular basis… he get sick often. It is like a routine.”*
	Struggle for access: Healthcare Disability certificate	*“We have to travel long distance to reach hospital; we have to start our journey early morning”* *“We did not get disability certificate because we had to make multiple visits to the hospital… the hospitals are also far”*
	Inconclusive diagnosis	*“We were not given any proper diagnosis so far. No one has told that this is the reason or diagnosis”*
	Lengthy hospital waits	*“I can wait for long time, but how will my child wait? He is uncontrollable.”*
	Poor social support	*“My husband does not take care of my son anymore… he is ashamed of him, so I have to take him to hospital alone”*
	Encircled by scammers	*“There are many people who try to reach us and manipulate us trying to sell unknown medicine which are very expensive. We used to buy thinking that my son will get well”*
	Discrimination	*“We did not apply for any schemes because people will discriminate my son”*
Facilitators for health seeking	Accessibility	*“If he is not feeling well, I immediately take him to the nearby PHC and get him checked”*
	Early intervention programs	*“I heard that there are health programmes that are available to find the disease early…. But I am not aware about it and I do not know where it is available.”*
	Access to information	*“We receive a lot of information from the special schools. They are very supportive and help us in many ways.”*
	Disability benefit	*“I am receiving benefit from the government. Along with that benefit, I use my earnings to manage the cost.”*
	Social support	*“My mother and sister support me. My family supports me a lot.”*
Recommendations from the parents	Improving the health system	*“It will be really helpful with community-based health checkup camps are conducted”* *“They should keep a separate reserved areas for handicapped people and give tablets.”* ***“**In government hospitals if there is a special ward, for these children, it will be good.”* *“If she is getting affected by anything, if the facilities are nearby, it will be easy for us.”* *“It will be helpful if they do not make us wait… Our children with autism run here and there”*
	Subsidiaries	*It will be very useful if rubber sheets, diapers are given at a subsided cost.”*

**Table 4:** Integration of quantitative and qualitative findings with meta-inferences using a joint display approach.

Overarching themes	Quantitative results	Qualtitative results	Metainferences
Households availing disability benefit	Among the households with differently abled children, approximately 55% did not receive a disability benefit.	Parents considered disability benefits as a channeling means for both livelihood and healthcare; however, there was difficulty in accessing them. *Supporting quotes: “My son is receiving pension amount, which is going to rent and his medical expenses,”* *“It was difficult to get the disability pension for my child. After 12 years only we are receiving the pension.”*	*Discordant:* though parents mentioned that the disability benefits were useful for them, the majority did not avail of the services due to reasons such as difficulty in accessing them.
Travel expense	The median (IQR) among those who faced CHE and those who did not were ₹4,400 (₹1,300, ₹10,100) and ₹1,000 (₹400, ₹3,900), respectively.	The participants expressed their plight due to the high travel burden. *Supporting quotes: “We cannot take him in two-wheeler, so we have to go by auto, bus or car.”* *“We had to travel up and down to Chennai and for per trip we had to spend 4.5K to get specialist opinion.”*	*Concordant:* the cost of travel was relatively high among households who were experiencing CHE compared to those who were not experiencing CHE.
Coping mechanisms	Households predominantly utilized borrowing (77%) as their primary coping strategy, followed by the sale of assets (47.9%), support from others (45.8%), delayed medical visits (33%), and extra work (29%).	The sale of assets, borrowing of money, delayed hospital visits and overtime work were expressed as the copind strategies by the parents. *Supporting quotes:* *“We sold all the jewels I had. So, we do everything by borrowing money from others only”* *“Borrowed money from my friends for around 5 lakhs because for per day check-up, we need 7,000 to 8,000 rupees”* *“Sometimes we delay hospital visits and checkups as we do not have adequate money.”*	*Concordant:* borrowing money was the most adopted coping strategy explaining the higher prevalence of distress financing.

## Discussion

The study assessed the prevalence of CHE among households with differently abled children/adolescents (44.8% at a 40% CTP threshold), estimated the additional monthly income required through CV analysis (median ₹3,000), and identified key facilitators and barriers influencing healthcare access through qualitative interviews. In this study, only 55.2% had a disability certificate, essential for accessing government benefits, with many reporting procedural delays and multiple doctor visits. Only 45% of households were availing disability benefits from the government and other sources. Our CHE prevalence is higher than that reported in a study among people with chronic illness in Bangalore by Swetha et al. (16), where 14.9% of households experienced CHE. At the national level, analysis of NSS 76th round data found that 57.1% of households with a disabled member faced CHE [Yadav et al. (17)]. It also slightly exceeded the findings of a study conducted in Iran by Moradi et al. (18). These patterns highlight disability as a major driver of household financial burden across diverse settings in India. OOP increased across wealth quintiles, aligning with existing literature showing higher inpatient utilization and OOP among wealthier groups (17, 19).

Significant disparities in travel expenses were observed between urban and rural households. Despite government provisions such as free bus transportation, challenges related to limited awareness and inadequate accessible transport options persist (20). A higher proportion of households reported distress financing, especially among lower-income groups who relied on borrowing, while income and savings were used by their counterpart (21). The CV analysis revealed that after propensity score matching, most households in group 1 had higher income levels, suggesting that additional income is required to maintain a comparable standard of living. Studies show that women’s participation in the labour market, hours worked, and income are adversely affected if they look after children with disabilities. Caring for a differently abled child was also found to affect the father’s income levels (22). Research conducted in other countries has also demonstrated a higher compensating variation, with the value tending to rise alongside the severity of the disability (23). Awareness and enrolment in the NIRAMAYA scheme were very low, consistent with the scenario in the general context (24).

The qualitative interviews unveiled a spectrum of barriers within the healthcare system, including limited access to services, financial burdens, and emotional distress linked to an inconclusive diagnosis. System-level challenges, such as poor continuity of care, long waiting times, and perceived disrespect from healthcare providers, further impede care seeking. At the societal level, discrimination, lack of support, and exposure to fraudulent practices promoting unnecessary treatments emerged as significant concerns. Experiences of disrespect deeply affected parents, fostering distrust in the healthcare system and influencing their overall engagement with services. On the other hand, facilitators within the health system included improved accessibility, availability of information, and supportive mechanisms.

External enablers such as government assistance and NGO support also play a critical role in easing the healthcare journey. Distrust in healthcare providers reported by several parents emerged as a barrier to both initial access and sustained engagement with the health system. Negative past experiences, perceived disrespect, and lack of transparent communication led some families to avoid follow-up visits or bypass certain facilities altogether. This distrust can disrupt continuity of care, delay timely interventions, and contribute to poorer long-term health outcomes, highlighting the need for provider training in disability-sensitive communication and patient-centered care.

These findings highlight the need for targeted interventions and policy reforms to address the complex barriers faced by these families, including financial strain, distrust in public healthcare, and lack of social support, as reflected in previous studies conducted in Cameroon and India (13, 25). Community engagement and social inclusion were the key facilitators of healthcare access. While government efforts are acknowledged, more comprehensive and sustained support is essential. The study is limited to potential recall and reporting bias, particularly for irregular expenses, and could have resulted in under- or over-estimation of the CHE prevalence; thus, these findings may not be generalized beyond the study setting. Given the cross-sectional design, this study cannot establish temporal or causal relationships. A higher precision value for sample size estimation, used due to logistical and time constraints, may have slightly reduced the accuracy of the prevalence estimates.

Given the low enrolment in social protection schemes, limited uptake of benefits, and the presence of a “missing middle” population, households with a differently abled child or adolescent who are ineligible for government health insurance yet unable to afford private coverage, targeted outreach through community health workers, Anganwadi centers, and school-based programs is essential (26). Simplifying disability certification procedures and linking them to automatic enrolment in public insurance schemes could improve coverage. Policy measures should include targeted premium subsidies, expansion of scheme benefits to cover rehabilitation and assistive devices, and addressing indirect costs such as transport through subsidies available in both rural and urban areas.

## Conclusion

The study found that a significant portion of households experiencing financial burden due to their child’s disability often resort to distress financing to manage the associated expenses. Limited coverage of disability benefits and other schemes was observed, with parents highlighting numerous hurdles in obtaining them. Various barriers to health seeking, such as financial constraints, lack of information, difficulties in accessibility, non-availability, distrust in the healthcare system, and lack of social support, were identified. On the other hand, facilitators such as good accessibility, availability of healthcare facilities, and good social support were also highlighted. Parents recommended key strategies related to health system strengthening and provision of subsidies to improve services for differently abled children.

## Data Availability

The raw data supporting the conclusions of this article will be made available by the authors, without undue reservation.

## References

[1] World Health Organization. Disability factsheet (2023). Available online at: https://www.who.int/news-room/fact-sheets/detail/disability-and-health (accessed January 29, 2023)

[2] Ministry of Statistics and Programme Implementation. Persons with disabilities (Divyangjan) in India: a statistical profile 2021. New Delhi: Government of India; (2021). Available online at: https://mospi.gov.in (accessed February 13, 2023)

[3] World Health Organization. Global health observatory: out-of-pocket expenditure estimates. Available online at: https://www.who.int/data/gho (accessed January 29, 2023)

[4] Selvaraj S Bhan N Mahal A. India health system review. Health Syst Transit (2022);11:1–325. Available online at: https://iris.who.int/handle/10665/352685 (accessed December 5, 2023)

[5] Joe W. Health insurance coverage in India: insights for National Health Protection Scheme. Indian J Hum Dev. (2018) 12:177–200.

[6] UNICEF. Ensure healthy lives and promote well-being for all at all ages: Universal health coverage. Available online at: https://data.unicef.org/wp-content/uploads/2018/04/SDG-briefing-note-6_universal-health-coverage.pdf (accessed December 5, 2023)

[7] UNICEF. SDG goal 3: good health and well-being. Available online at: https://data.unicef.org/sdgs/goal-3-good-health-wellbeing/ (accessed June 20, 2024)

[8] Filmer D. Disability, poverty, and schooling in developing countries: results from 14 household surveys. World Bank Econ Rev. (2008) 22:141–63. doi: 10.1093/wber/lhm021

[9] Government of Puducherry. Schemes for disabled. National Interactive Web Portal. Available online at: https://www.py.gov.in (accessed February 13, 2023)

[10] Solmi F Melnychuk M Morris S. The cost of mental and physical health disability in childhood and adolescence to families in the UK: findings from a repeated cross-sectional survey using propensity score matching. BMJ Open. (2018) 8:e019209. doi: 10.1136/bmjopen-2017-018729, PMID: 29391378 PMC5829601

[11] Mishra S Mohanty SK. Out-of-pocket expenditure and distress financing on institutional delivery in India. Int J Equity Health. (2019) 18:83. doi: 10.1186/s12939-019-1001-7, PMID: 31238928 PMC6593606

[12] Wapling L Schjoedt R Sibun D. Social protection and disability in India. Development Pathways (2019). Available online at: http://www.developmentpathways.co.uk (accessed December 5, 2023)

[13] Zuurmond M Mactaggart I Kannuri N Murthy G Oye JE Polack S. Barriers and facilitators to accessing health services: a qualitative study amongst people with disabilities in Cameroon and India. Int J Environ Res Public Health. (2019) 16:1126. doi: 10.3390/ijerph16071126, PMID: 30934813 PMC6480147

[14] Agbelie CM. Health system access challenges of people with disabilities increased during COVID-19 pandemic (2023). Available online at: https://www.who.int/publications (accessed March 22, 2024) 10.1016/j.dhjo.2023.101446 PMC9877151 36804186

[15] Haakenstad A Kalita A Bose B Cooper JE Yip W. Catastrophic health expenditure on private sector pharmaceuticals: a cross-sectional analysis from the state of Odisha, India. Health Policy Plan. (2022) 37:872–84. doi: 10.1093/heapol/czac035, PMID: 35474539 PMC9347020

[16] Swetha NB Shobha S Sriram S. Prevalence of catastrophic health expenditure and its associated factors, due to out-of-pocket health care expenses among households with and without chronic illness in Bangalore, India: a longitudinal study. J Prev Med Hyg. (2020) 61:E92–7. doi: 10.15167/2421-4248/jpmh2020.61.1.1191 32490274 PMC7225654

[17] Yadav J Tripathi N Menon GR Nair S Singh J Singh R. Measuring the financial impact of disabilities in India: an analysis of national sample survey data. PLoS One. (2023) 18:e0293437. doi: 10.1371/journal.pone.0292592 PMC10569625 37824482

[18] Moradi G Bolbanabad AM Abdullah FZ Safari H Rezaei S Aghaei A. Catastrophic health expenditures for children with disabilities in Iran: a national survey. Int J Health Plann Manag. (2021) 36:1861–73. doi: 10.1002/hpm.3273, PMID: 34185916

[19] Mohanty SK Singh S Sharma SK Banerjee K Acharya R. Asset and consumption gradient of health estimates in India: implications for survey and public health research. J Econ Inequal. (2022) 20:675–701. doi: 10.1016/j.ssmph.2022.101258 PMC9550646 36238815

[20] Clemente KAP da Silva SV Vieira GI de Bortoli MC Toma TS Ramos VD. Barriers to the access of people with disabilities to health services: a scoping review. Rev Saude Publica (2022);56:43. Available online at: https://www.scielo.br/j/rsp (accessed June 24, 2024) 35792776 10.11606/s1518-8787.2022056003893 PMC9239543

[21] Sangar S Dutt V Thakur R. Distress financing of out-of-pocket health expenditure in India. Rev Dev Econ. (2019) 23:314–30. doi: 10.1111/rode.12540

[22] Yisfashewa Wondemu M Hermansen Å Brekke I. Impact of child disability on parental employment and labour income: a quasi-experimental study of parents of children with disabilities in Norway. BMC Public Health. (2021) 21:2113. doi: 10.1186/s12889-022-14195-5 36151541 PMC9508753

[23] Van Minh H Giang KB Liem NT Palmer M Thao NP Duong LB. Estimating the extra cost of living with disability in Vietnam. Glob Public Health. (2015) 10:S70–9. doi: 10.1080/17441692.2014.971332 25353274

[24] Angothu H Ajmera S Thanapal S Reddy K Jagannathan A Muliyala K. Poor enrollment of persons with disabilities in Niramaya health insurance scheme over a decade under the Indian National Trust. Indian J Soc Psychiatry. (2022) 38:297–300. doi: 10.4103/ijsp.IJSP_189_20

[25] Foo KM Sundram M Legido-Quigley H. Facilitators and barriers of managing patients with multiple chronic conditions in the community: a qualitative study. BMC Public Health. (2020) 20:273. doi: 10.1186/s12889-020-8375-8, PMID: 32106838 PMC7045577

[26] Kumar A Sarwal R. Health insurance for India’s missing middle. NITI Aayog (2021). Available online at: https://www.niti.gov.in/sites/default/files/2023-02/Health-Insurance-for-India%E2%80%99s-Missing-Middle_08-12-2021.pdf (accessed December 5, 2023)

